# Unilateral *BEST1*-Associated Retinopathy

**DOI:** 10.1016/j.ajo.2016.05.024

**Published:** 2016-09

**Authors:** Rashi Arora, Kamron Khan, Melissa L. Kasilian, Rupert W. Strauss, Graham E. Holder, Anthony G. Robson, Dorothy A. Thompson, Anthony T. Moore, Michel Michaelides

**Affiliations:** a Moorfields Eye Hospital, London, United Kingdom; b Salisbury District Hospital, Salisbury, United Kingdom; c University College London Institute of Ophthalmology, London, United Kingdom; d Great Ormond Street Hospital, London, United Kingdom; e Department of Ophthalmology, University of California San Francisco School of Medicine, San Francisco, California

## Abstract

**Purpose:**

To describe a series of patients with molecularly confirmed mutation in *BEST1* causing Best disease but with unilateral clinical manifestation.

**Design:**

Retrospective observational case series.

**Methods:**

Setting: Moorfields Eye Hospital and Great Ormond Street Hospital, London (United Kingdom). Patients: Five patients (10 eyes) with uniocular manifestation of *BEST1* mutation causing Best disease were ascertained retrospectively from the clinical and genetic databases. Main Outcome Measures: Patients had full ophthalmologic examination, color fundus photography, fundus autofluorescence imaging, spectral-domain optical coherence tomography, and detailed electrophysiological assessment. Genetic testing was performed.

**Results:**

All cases had a clinical appearance typical of and consistent with Best disease at various stages, except that the presentation was unilateral. The reduced electrooculogram light rise was bilateral and in the context of normal electroretinograms therefore indicates generalized dysfunction at the level of the retinal pigment epithelium.

**Conclusions:**

Mutation in *BEST1* has variable penetrance and expressivity, and can be uniocular. The clinical and electrophysiological features described assist targeted mutational screening and alert to the potential diagnosis even when there is an atypical unilateral presentation.

Best disease was first described by Adams in 1883, but was named after Dr Friedrich Best, who identified an autosomal dominant mode of inheritance after examining 7 members of a pedigree segregating this disorder.^1^ Best disease (vitelliform macular dystrophy) is an early-onset macular dystrophy typically characterized by bilateral accumulation of subretinal deposit resulting from heterozygous mutations in the *BEST1* gene (OMIM 153700). It is a slowly progressive macular dystrophy with usual onset in childhood but sometimes in later teenage years. The classic appearance of yolk-like lesions is a striking feature and distinguishes it from other hereditary conditions. The phenotype can vary significantly, even within the same family. The most extreme example of this is nonpenetrance of the macular changes in the presence of electrophysiological evidence of disease. The retinal changes are typically bilateral and relatively symmetrical, but rarely, inherited *BEST1* mutations may be associated with unilateral maculopathy, with only 3 cases reported in the literature to date.^1^, ^2^ The present report describes a series of 5 molecularly proven cases with unilateral presentation of Best disease.

## Methods

Patients were ascertained retrospectively from the clinical and genetic databases of Moorfields Eye Hospital, London, United Kingdom and Great Ormond Street Hospital, London, United Kingdom. Patients and family members received full ophthalmologic examination including visual acuity testing using Snellen charts, color fundus photography, fundus autofluorescence imaging, and spectral-domain optical coherence tomography (Heidelberg Engineering, Heidelberg, Germany). Electrophysiological assessment included full-field and pattern electroretinography and electrooculography.^3^, ^4^, ^5^ Blood samples were taken for DNA extraction and mutation screening of *BEST1* by Sanger sequencing. The study was approved by the local ethics committee of Moorfields Eye Hospital. All patients, or their parents, gave informed consent and the study conformed to the tenets of the Declaration of Helsinki and complied with the Health Insurance Portability and Accountability Act.

## Results

### Family 1 (Cases 1 and 2)

**Figure 1:**
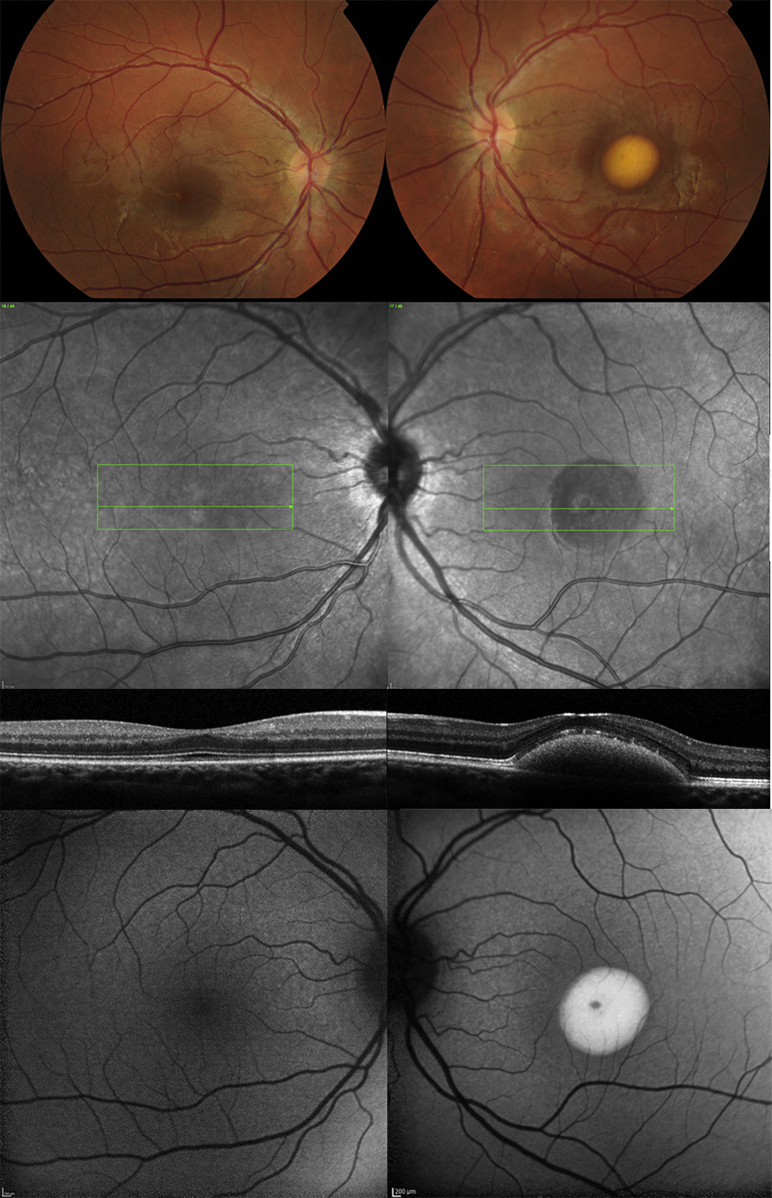
Multimodal imaging of (Left column) the right eye and (Right column) the left eye of patient (Case 1) with unilateral *BEST1*-associated retinopathy. Color fundus photographs (Top row), infrared reflectance images (Second row), horizontal B-scans derived from spectral-domain optical coherence tomography through the foveal region (Third row), and fundus autofluorescence images (Bottom row) of both eyes are shown. The left eye presents with a typical yolk-like elevated lesion at the central macula that was hyperautofluorescent on fundus autofluorescence; spectral-domain optical coherence tomography revealed subretinal fluid in addition to the subretinal deposit.

**Figure 2:**
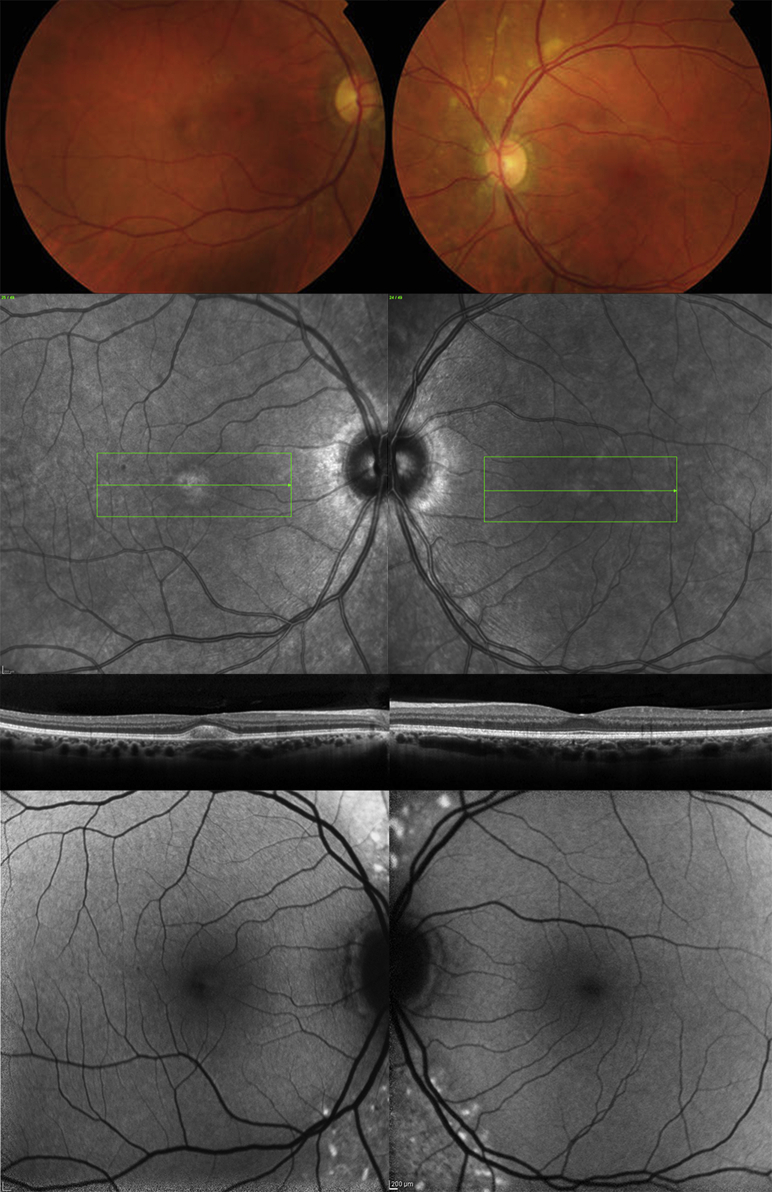
Multimodal imaging of (Left column) the right eye and (Right column) the left eye of patient (Case 2; mother of patient in Case 1) with unilateral *BEST1*-associated retinopathy. Color fundus photography (Top row), infrared reflectance imaging (Second row), horizontal B-scan through the foveal region by spectral-domain optical coherence tomography (Third row), and fundus autofluorescence (Bottom row) are presented. Subretinal deposit as detected by spectral-domain optical coherence tomography was present only in the right macula. Fundus autofluorescence showed bilateral, relatively symmetrical areas of increased autofluorescence in the nasal retina.

A 12-year-old boy (Case 1, Table) presented to the eye clinic with recent onset of blurred distance vision in the left eye. Best-corrected logMAR visual acuity was 0.02 in the right eye and 0.06 in the left. Near vision was N5 in each eye and no distortion was reported using an Amsler grid. There was a family history of macular dystrophy affecting his grandmother, who was registered blind, and his maternal uncle, who maintained driving vision. Funduscopy of the left eye showed a yolk-like elevated lesion at the central macula that was hyperautofluorescent on fundus autofluorescence imaging (Figure 1). Spectral-domain optical coherence tomography revealed subretinal fluid in addition to the subretinal deposit. Fundus examination, fundus autofluorescence, and spectral-domain optical coherence tomography of the right eye were normal. His mother (Case 2, Table) had similar uniocular features on funduscopy at the posterior pole of the right eye but was asymptomatic. Fundus autofluorescence showed bilateral, relatively symmetrical areas of increased autofluorescence in the nasal retina, but spectral-domain optical coherence tomography abnormality was present only at the right macula (Figure 2). Electrophysiological testing showed absence of the electrooculogram light rise in both eyes of both mother and son. Full-field electroretinography was normal in both patients. Pattern electroretinograms were within normal limits in both eyes of the son, showing minimal interocular difference with lower amplitudes in the left eye using a 15-degree field stimulus. Pattern electroretinography was not performed in Case 2 (mother). In both patients, genetic testing identified a previously reported heterozygous sequence variant, c.692G>C, p.Ser231Thr, in *BEST1*.^6^

### Case 3

**Figure 3:**
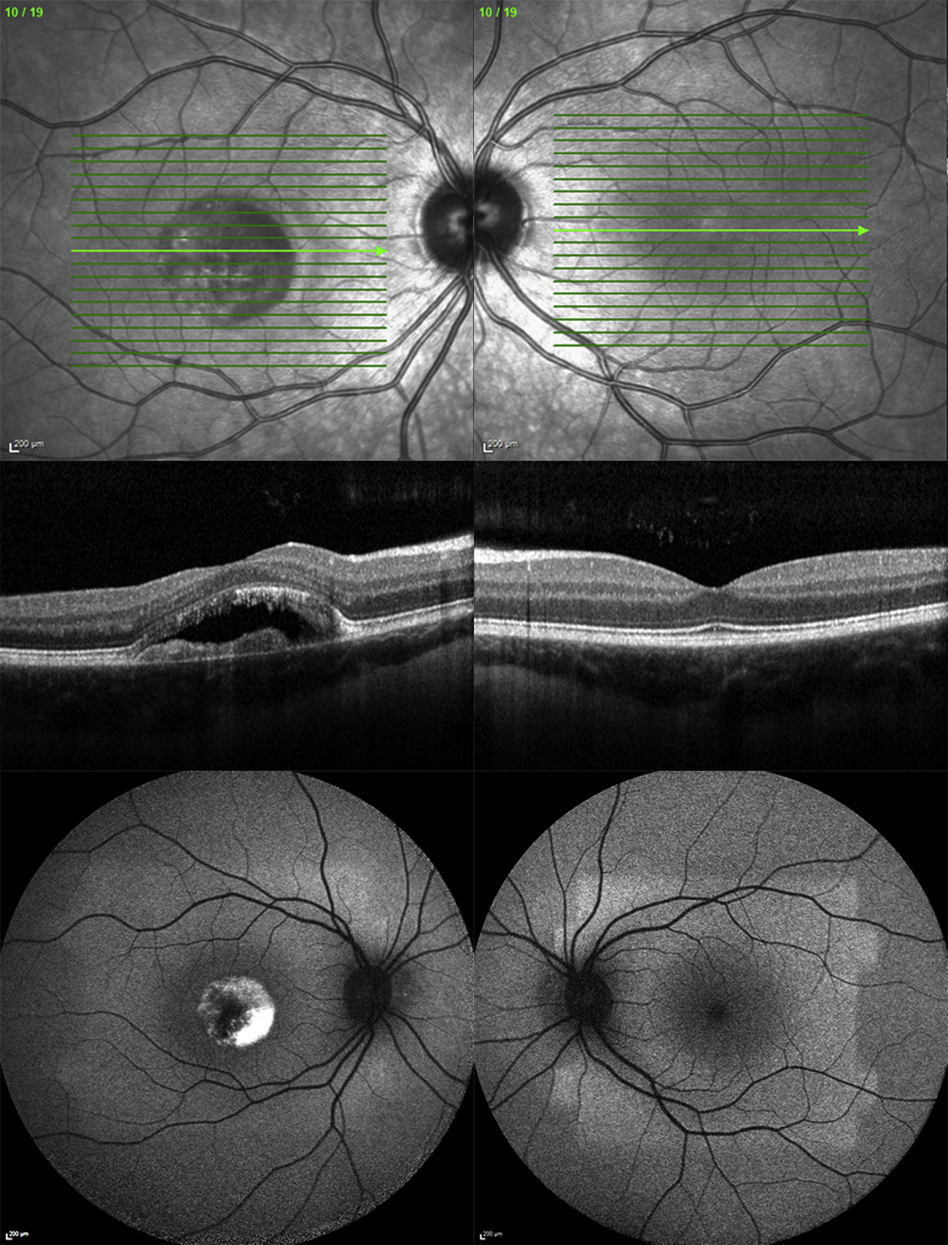
Multimodal imaging of (Left column) the right eye and (Right column) the left eye of patient (Case 3) with unilateral *BEST1*-associated retinopathy. Infrared reflectance imaging (Top row), horizontal B-scan through the foveal region by spectral-domain optical coherence tomography (Middle row), and fundus autofluorescence (Bottom row) are shown. The right eye shows macular atrophy, yellow subretinal and subretinal pigment epithelium deposition, and subretinal fluid.

A 16-year-old male subject presented with reduced vision in his right eye (Table). His father had been diagnosed with Best disease at an early age based on the presence of bilateral vitelliform lesions, but remained asymptomatic until the age of 30. Two paternal aunts were known to have Best disease. Best-corrected logMAR visual acuity was -0.1 in the right eye and 0.0 in the left. Funduscopy, fundus autofluorescence, and spectral-domain optical coherence tomography of the right eye revealed macular atrophy, yellow subretinal and subretinal pigment epithelium deposition, and subretinal fluid (Figure 3). The left eye was normal on funduscopy and imaging. The pattern electroretinogram was significantly reduced in the right eye but normal in the left eye. Full-field electroretinograms were normal in both eyes. Electrooculogram light rise was subnormal in both eyes. *BEST1* screening identified that both father and son were heterozygous for a previously reported sequence variant, c.47C>T, p.Ser16Phe, in exon 2 of *BEST1*.^7^

### Case 4

**Figure 4:**
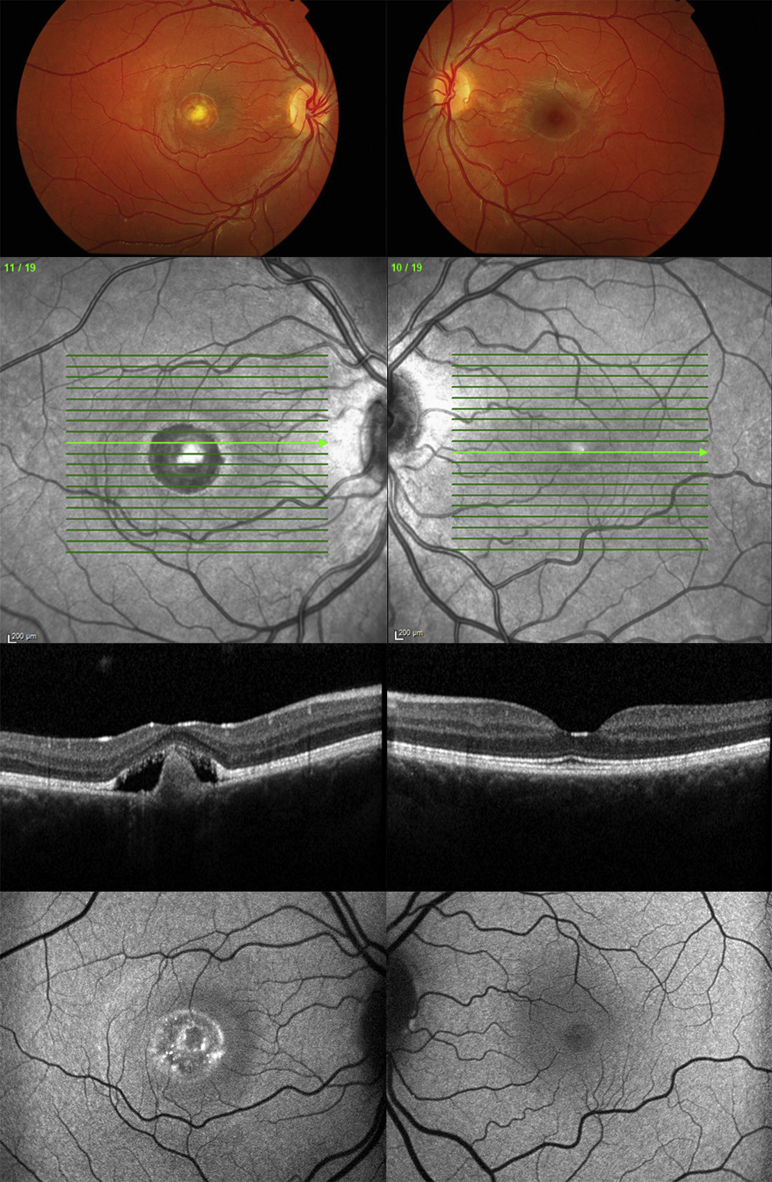
Multimodal imaging of (Left column) the right eye and (Right column) the left eye of patient (Case 4) with unilateral *BEST1*-associated retinopathy. Color fundus photographs (Top row), infrared reflectance images (Second row), horizontal B-scans derived from spectral-domain optical coherence tomography through the foveal region (Third row), and fundus autofluorescence images (Bottom row) of both eyes are presented. The right eye shows vitelliform changes associated with increased autofluorescence on fundus autofluorescence and subretinal and subretinal pigment epithelium deposition on spectral-domain optical coherence tomography.

A 17-year-old asymptomatic female patient was found by her optometrist to have an abnormal appearance of the right macula (Table). There was no known family history of eye disease. Best-corrected logMAR visual acuity was 0.06 in the right eye and 0.0 in the left. Fundus examination showed unilateral vitelliform changes in the right eye associated with increased autofluorescence on fundus autofluorescence and subretinal and subretinal pigment epithelium deposition on spectral-domain optical coherence tomography (Figure 4). The left eye was normal on funduscopy and multimodal imaging. Her father was asymptomatic and funduscopy was normal. However, fundus autofluorescence imaging revealed hyperautofluorescent subretinal deposits in both eyes. Both father and daughter had significantly reduced electrooculogram light rise in both eyes, consistent with Best disease. In both the father and the daughter, *BEST1* screening identified a previously reported heterozygous disease-causing mutation c.874G>A, p.Glu292Lys, in exon 8 of *BEST1*.^8^

### Case 5

**Figure 5:**
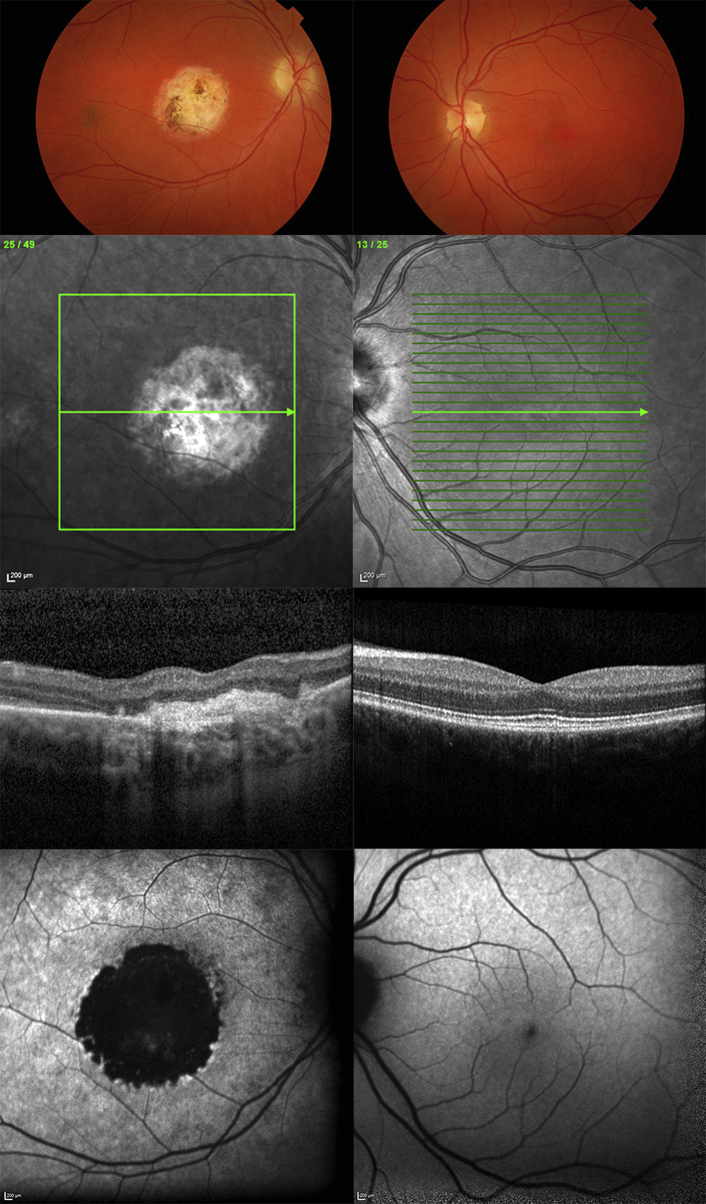
Multimodal imaging of (Left column) the right eye and (Right column) the left eye of patient (Case 5) with unilateral *BEST1*-associated retinopathy. Color fundus photographs (Top row), infrared reflectance images (Second row), horizontal B-scans derived from spectral-domain optical coherence tomography through the foveal region (Third row), and fundus autofluorescence images (Bottom row) of both eyes are presented. In color fundus photography, there is a macular scar in the right eye that corresponds to a large area of macular hypoautofluorescence on fundus autofluorescence imaging and subretinal fibrosis on spectral-domain optical coherence tomography.

A 27-year-old male patient was reviewed with longstanding poor vision in the right eye (Table). Best-corrected logMAR visual acuity was hand movements in the right eye and 0.0 in the left. Fundus examination showed a macular scar in the right eye, but was normal on the left. Fundus autofluorescence imaging showed a large area of macular hypofluorescence and corresponding area of subretinal fibrosis on spectral-domain optical coherence tomography (Figure 5). Full-field electroretinograms were normal bilaterally. Unfortunately, electrooculography was technically unsatisfactory on 2 occasions. His mother and sister were reported to be affected but family members were not available for examination. Genetic testing revealed a heterozygous previously reported variant in *BEST1*, c.892T>G, p.Phe298Val. Other mutations of codon 298 have previously been reported.^9^, ^10^

## Discussion

This report describes 5 patients with uniocular clinical manifestation of disease-causing variants in *BEST1* (Best disease). The clinical features were otherwise typical of the disorder. Three patients presented between 12 and 17 years of age with good visual function. In 2 cases a parent was identified as being affected despite being asymptomatic, and in the final case the father was already known to have bilateral Best disease.

All cases had appearance typical and consistent with Best disease except that the presentation was unilateral. The fundus autofluorescence and spectral-domain optical coherence tomography imaging demonstrated features of different stages of Best disease. The reduced electrooculogram light rise was bilateral and, in the context of normal electroretinograms, therefore indicates generalized dysfunction at the level of the retinal pigment epithelium. An abnormal electrooculogram light rise has been previously reported in *BEST1* carriers with normal fundus appearance and can precede the clinical manifestation of vitelliform lesions.^11^, ^12^ While full-field electroretinograms were within normal limits in all patients, pattern electroretinograms were variable in patients in whom testing was undertaken: normal in Case 1 (although slight interocular asymmetry to a 15-degree field) and reduced in the “affected” eye of the patient in Case 3 compared to normal in the fellow eye.

It is known that Best disease can have reduced penetrance and variable expression, but the mechanisms are poorly understood.^1^, ^9^, ^13^, ^14^, ^15^, ^16^, ^17^, ^18^, ^19^ Variable expressivity is common in Best disease even within families that carry the same causative mutation; this is likely to be because of the influence of modifier genes. Most individuals diagnosed with Best disease have an affected parent; however, in autosomal recessive bestrophinopathy, where patients harbor biallelic variants in *BEST1*, that is not the case.^20^ The proportion of dominant cases caused by de novo mutations is unknown.

*BEST1* encodes the transmembrane protein bestrophin 1, which is located on the basolateral aspect of the plasma membrane of retinal pigment epithelium cells. There are over 200 mutations described in *BEST1* (http://www.retina-international.org/sci-news/databases/mutation-database/best1-mutation/, last accessed March 5, 2016). Although the encoded protein's exact function remains incompletely described, it has been linked to abnormal chloride conductance, which likely disrupts fluid transport across the retinal pigment epithelium and leads to accumulation of debris between Bruch's membrane and the retinal pigment epithelium/photoreceptor complex.^21^, ^22^, ^23^ However, the colocalization of bestrophin 1 and STIM1 has been reported, and an association with the endoplasmic reticulum and cytosolic compartment next to the basolateral membrane, which suggests bestrophin is also involved in modulation of intracellular Ca2+ storage, in addition to acting as a Ca2+-activated Cl− channel, and provides an explanation of why some patients with Best disease have a normal light rise.^24^, ^25^

Singh and associates have shown that rhodopsin degradation after photoreceptor outer segment feeding was delayed in induced pluripotent stem cell–derived retinal pigment epithelium cells from patients with Best disease compared with induced pluripotent stem cell–derived retinal pigment epithelium cells from unaffected siblings, directly implicating impaired photoreceptor outer segment handling in the pathophysiology of the disease.^26^ In addition, stimulated calcium responses differed between Best disease and normal sibling induced pluripotent stem cell–derived retinal pigment epithelium, as did oxidative stress levels after chronic photoreceptor outer segment feeding.^26^

Best disease is almost always bilateral.^2^ However, 2 patients have been reported by Querques and associates with unilateral Best disease and heterozygous mutations in exon 2 of *BEST1*, unrelated either to their age or to their genotype.^2^ One of these patients, aged 27, shared the p.T4A mutation with his 23-year-old sibling, who had bilateral disease (CT08). Similarly, the second patient, a 70-year-old man, had 2 affected family members (aged 10 and 36 years) with bilateral disease that carried the same p.R25W mutation. Wabbels and associates reported a 7-year-old boy with unilateral disease associated with the p.Ile295del mutation, which is normally associated with bilateral disease.^1^

This series of 5 patients supports that *BEST1* mutation causing Best disease can have a uniocular clinical manifestation, with otherwise typical clinical and imaging features. The most consistent phenotypic feature, even in Best disease cases with stage 1 disease and a normal macular appearance, is a reduced light rise of the electrooculogram. Electrooculography was bilaterally abnormal in the present series, indicating generalized retinal pigment epithelium dysfunction in each eye but with abnormal macular structure only in 1 eye, stressing the diagnostic importance of electrooculography. The cause of this unilateral presentation, rare in other inherited retinal dystrophies, is currently unknown.

## Figures and Tables

**Table:** Patient Characteristics and Demographic and Genetic Information for the 5 Patients With Unilateral *BEST1*-Associated Retinopathy

Case	Patient Age at Presentation (y)	Sex	Visual Acuity at Presentation (logMAR) in the Affected Eye	Fundus Phenotype of the Affected Eye	Fundus Phenotype of the Fellow Eye	Electrooculography Light Rise	Parental Presentation	Disease-causing Mutation in the *BEST1* Gene
1	12	Male	0.06	Creamy elevated lesion in central macular area	Normal	Absent light rise both eyes	Mother carrier state, unilateral	c.692G>C, p.Ser231Thr
2 (Mother of Case 1)	38	Female	0.0	Faint yellowish spot in the right fovea	Normal	Absent light rise both eyes	Mother (grandmother of Case1) with bilateral macular dystrophy	c.692G>C, p.Ser231Thr
3	16	Male	−0.1	Macular atrophy with some yellow deposits at the level of the RPE	Normal	Reduced light rise both eyes	Father with known Best disease, bilateral	c.47C>T, p.Ser16Phe,
4	17	Female	0.06	Vitelliform lesion	Normal	Bilateral reduction	Father carrier state, bilateral	c.874G>A, p.Glu292Lys
5	27	Male	Hand motions	Atrophic lesion	Normal	Technically unsatisfactory	Mother and sister affected, although family members not examined	c.892T>G, p.Phe298Val

RPE = retinal pigment epithelium.
